# The psychosomatic experiences of women who had intrauterine foetal death in rural South Africa

**DOI:** 10.4102/curationis.v46i1.2279

**Published:** 2023-12-05

**Authors:** Martha Kharivhe, Mary Maluleke, Thingahangwi Masutha, Takalani Thabathe, Duppy Manyuma, Ndivhaleni Lavhelani, Muofheni Nemathaga, Muvhango Ramovha, Mutshinyalo Netshikweta, Mulatedzi Mulaudzi

**Affiliations:** 1 Department of Advance Nursing, Faculty of Health, University of Venda, Thohoyandou, South Africa

**Keywords:** experiences, intrauterine foetal death, labour, mind–body, women

## Abstract

**Background:**

Intrauterine foetal death (IUFD) is a traumatic event leading to substantial grief reactions with a variety of experiences in an expectant woman. After delivery, these experiences have shown to impact the mother’s psychological well-being, where she experiences post-traumatic stress, sadness, anxiety and depression. The psychosomatic experiences before labour commenced are not known.

**Objectives:**

This study explored the psychosomatic (mind–body connection) experiences of women who had an IUFD before labour commenced in rural areas of Limpopo province, South Africa.

**Method:**

A qualitative approach with an explorative descriptive design was carried out among all 10 consented participants who were selected using a purposive sampling technique. The sample consisted of women who delivered an IUFD as reflected by the hospital register from the selected hospitals. Data were collected at the participants’ homes through in-depth individual interviews guided by one open-ended central question as follows, ‘Please share with me your experiences of IUFD before you went into labour’, and analysed using Tesch’s open coding method.

**Results:**

Two themes reflecting the psychosomatic (mind–body connection) experiences of women who had an IUFD emerged from the analysis. The themes are danger alerts and emotional responses.

**Conclusion:**

This qualitative study revealed that women could relate a lack of or decreased foetal movement as the danger alert or warning sign that the baby was in danger before labour commenced. Upon noticing that something was wrong with the baby, a message was sent to the women’s minds, which equally affected and activated their emotional dimensions. An investigation regarding the kind of support needed by women after being informed of an IUFD is recommended.

## Introduction

Intrauterine foetal death (IUFD) is a common and invisible problem, but it is avoidable through managed care and enhanced information (Lawn, Cousens & Zupan 2015). Globally, IUFD leads to extensive sorrowfulness, sadness, feelings of blame, resentment and confusion in women following a diagnosis of IUFD (Costin & McMurrich 2015; Gausia et al. 2017). Although the responses might vary, they are even more intense following recurrent losses, and the anguish is aggravated by feelings of worry, disappointment and self-blame (Costin & McMurrich 2015). Hassan, Rothert and Saffran (2013) found that women felt supported and not alone when the medical staff kept them informed as problems arose.

In contrary, Boyle et al. (2018) discovered that women who had IUFD suffered from acute and chronic anxiety as well as depression related to the loss. In agreement, Pullen, Golden and Cacciatore (2018) allude that a mother’s grief after IUFD is further complicated by feelings of anxiety, failure and guilt, which complicates to post-traumatic stress disorder and psychosis.

Meanwhile, Honikman et al. (2014) in a case study report that the common mental disorders, namely prenatal and postpartum depression, anxiety and postpartum psychosis are mostly experienced by women who had IUFD from disadvantaged communities. In another study, the World Health Organization (WHO) (2018) reports that the consequences of IUFD differ depending on the personal and cultural values of a woman.

The Saving Mothers Report (2017) indicates that the prevalence of mental health problems experienced by women after IUFD is almost 40% in South Africa. In agreement, Koopmans et al. (2018) and Hull (2019) report that the IUFD experience is filled with tragedy, mourning and despair that causes major emotional problems to the grieving woman.

The phenomenon of the experiences of women who had IUFD has been widely studied. The struggle of whether to disclose the IUFD or not is echoed in the literature by the grieving mothers who experienced emotional pain, shame and guilt (Gopichandran, Subramaniam & Kalsingh 2018; Keeble & Thorsteinsson 2018). Furthermore, they experience social isolation and exclusion from friends, loved ones and family (Brierley-Jones et al. 2015; Cacciatore 2010; Cheer 2016; Thompson 2013).

In the same vein, the grieving mothers and their partners perceived IUFD to be unexpected, confusing and a frustrating experience because the precise cause was not clearly explained to them. As a result, they made their own conclusions, which include superstitions, medical negligence and blamed various persons in their lives for the occurrence (Gopichandran, Subramaniam & Kalsingh 2018; Sinaga, Purwarini & Anggraeni 2020; Sun, Rei & Sheu 2014).The latitude and magnitude of the mind–body experience of women is unclear. We must learn more and understand these experiences from all stages to assure sustainable provision of comprehensive and compassionate nursing care for women experiencing IUFD. Therefore, this study explored the psychosomatic (mind–body) experiences of women who had an IUFD before labour commenced in rural areas of Limpopo province, South Africa.

## Methods

### Study setting

This study occurred in Vhembe district, which is one of the five districts of Limpopo province in South Africa. Vhembe district is predominantly rural, with six district hospitals and one regional hospital. District hospitals are Level 1 hospitals, operating 24 h maternity services, staffed by midwives, advanced midwives and doctors. During the period from June 2015 to November 2015, a single district hospital registered a total number of 34 IUFD from the total deliveries of 1687; that is, there were 1653 live births (LB), equivalent to a rate of 20.1/1000 LB. Participants’ names were drawn from the three selected hospitals’ delivery registers and coded, to ensure participants’ confidentiality and anonymity.

### Research approach

A qualitative approach with an explorative, descriptive and contextual design was used. The approach was suitable because it could be used to see the perspectives of the participants (Polit & Beck 2017). All sampled women who had IUFD were allowed an opportunity to narrate their mind–body lived experiences. This study was contextualised as per the topic, the purpose, population and the setting, to accurately explore and describe the psychosomatic (mind–body connection) experiences of women who had IUFD before labour commenced as described by Polit and Beck (2017) and Grove and Gray (2019).

### Sampling method

Purposive sampling was done to select the district, the hospitals and participants. Vhembe District was sampled since it had the highest numbers of perinatal deaths (stillbirths & early neonatal deaths), and contributes 25% of total deliveries in the Limpopo province. Statistics SA 2012 recorded a total number of 8085 IUFD from 232 718 LB (34/1000 LB) in Limpopo province. Out of the seven hospitals in Vhembe District, three hospitals that had a high rate of IUFD, which was above 20/1000 LB, were purposively selected.

The study population were women who delivered IUFD as reflected by the district hospital maternity register from the selected hospitals. They were screened for eligibility to participate in this study based on the criteria of mothers who had experienced IUFD, 6 months from the time of the loss, as described by Videbeck (2011). Therefore, the inclusion criteria were women who delivered via normal vaginal delivery (NVD) between January 2019 and June 2019; 18 years and above; with no record or history of mental health problems; complete details such as the address and the telephone numbers; and gave consent voluntarily. The were included regardless of parity, marital status, religious background, level of education or employment status.

The exclusion criteria were NVD before January 2019 and after June 2019; delivered with caesarean section; below 18 years; with a history of mental health problems; incomplete details, such as the address and the telephone numbers; and did not give consent to participate in the study.

Purposive sampling, as described in Brink (2012), was adopted to select women who delivered IUFD as reflected by the hospital register from the selected hospitals. The total number of potential participants who met the study criteria were 16, and were all contacted and recruited telephonically. Out of 16, 10 voluntarily consented to participate in the study.

### Data collection

Upon arriving at each participant’s home, the interviews began with the greetings, which were then followed by an introduction and the telephonic agreement of the appointment. The research objectives and process were again explained to the participants, along with ethical considerations. Participants were given the opportunity to withdraw if they wished to do so.

Data were collected from women aged between 18–42 years and at 1st–6th pregnancy at the time of the IUFD, using an in-depth individual interview with all 10 consented participants. An in-depth individual interview was preferred as a data collection tool because it is a professional conversation that seeks deep information as well as an understanding of lived experiences from the interviewees’ perspectives (Grove & Gray 2019). Individual interviews were conducted in the Tshivenda language, as preferred by the participants, during the period from October 2019 to November 2019. The interview departed from the one central question, namely, ‘Please share with me your experiences of IUFD before you went into labour’.

The central question was asked to all participants as a point of departure, and then the probing questions were asked depending on the participants’ responses to the central question. The questions explored more deeply the women’s responses to retell what they experienced before labour commenced. Effective communication skills such as listening attentively, probing for more, linking and summarising the information narrated, nodding the head and saying ‘mmm’ for more details until the lived psychosomatic experience before labour commenced was thoroughly narrated and described.

The interviews were conducted with all 10 participants until no more new information emerged, for each participant. Therefore, interview was concluded when there was repetition of narrations to each participant. Next, the researchers and independent coder were satisfied that data saturation was achieved. The interview time ranged from approximately 45–60 min. All interviews were audio-recorded, transcribed verbatim and anonymised. Thereafter, translation from Tshivenda to English was done by the language expert for analysis.

### Data analysis

Collectively, all authors used the qualitative content of the Tesch open coding method of Cresswell and Cresswell (2014) to analyse the transcripts. To increase trustworthiness, the authors read the transcripts more than once to get an understanding of what participants said. Data were then arranged into themes and subthemes using the women’s actual words. Similar categories were allocated to a specific theme that captured the same idea. Grounded theory in Polit and Beck (2017) of thematic content was used as a guide to address interpretation differences until consensus was reached. Two researchers who are PhD holders and qualitative research experts collectively coded the findings, guided by Polit and Beck (2017). Finally, two themes and six subthemes emerged which were learned regarding the mind–body experiences of women who had IUFD before labour commenced.

### Ethical considerations

Ethical measures were observed throughout the study to protect the rights of the participants, as described by Polit and Beck (2017) and Grove and Gray (2019). The study was approved in 20 May 2019 by the Higher Degrees Ethics Committee and Research and Publications Committee of the University of Venda (reference number SHS/19/PDC/09/1405). Permissions were granted by the Limpopo Provincial Department of Health (25 July 2019) and the Vhembe District Department of Health (02 August 2019). Furthermore, permissions were granted by chief executive officers of the selected hospitals, respectively, to access clinical records of women above 18 years who had IUFD.

During the telephone recruitment of participants, a full explanation of the study was narrated, and the appointments were made regarding the place and time convenient to participants. All participants preferred their homes as a place where interviews should take place to ensure privacy. During the first meeting with each participant at their homes, once more a full explanation of the study was provided to participants. The participants were informed that they were under no obligation to participate in the study and that they could withdraw from the study at any point. An information pamphlet about the study and the contact numbers of supervisors was provided to all the participants in Tshivenda and English, in case they needed clarity to ensure voluntary participation. All participants gave informed consent, where eight gave written consent by signing, and two gave verbal consent, as they indicated being uncomfortable with appending their signatures. Furthermore, permission to use the audio recorder to record the interviews was sought from participants, and they were shown a stop button to discontinue recording at any time they wished. During transcribing and reporting, coding of participants (where each participant was given an identifiable code) and refraining from naming villages and hospitals was used to ensure anonymity and confidentiality.

Since the content of the study was emotional and could unearth the negative emotions where a woman might relapse and re-experience the emotional trauma, a backup plan was in place as follows: the study team members consisted of five advanced psychiatric nurses and two clinical psychologists who are mental health expects. Thus, they were available to intervene in times of emotional relapse by women during the study. Secondly, the first author is a psychiatric nurse with knowledge of the signs of a mental health problem and could offer intervention and refer accordingly.

### Trustworthiness

Trustworthiness, as outlined in Lincoln and Guba (1985) and De Vos et al. (2014), was observed. Credibility was enhanced through prolonged engagement with participants telephonically during recruitment, physically during data collection and member checking. In some instances, questions were repeated for more clarity. Participants were permitted to express their experiences without being interrupted. With each participant, interviews were conducted until she had nothing new to say. The credibility of the data was achieved through reading the manuscripts multiple times and discussing themes in a series of meetings and the three authors’ independent generation of theme and subthemes. Discrepancies were resolved through several discussions until consensus was reached.

To ensure dependability, cross-checking of codes was allowed, for other qualitative research experts to see whether the experts would code the same way as the researcher, from authors with diverse levels of experience with qualitative data analysis from their professional backgrounds.

Confirmability guaranteed that the findings, conclusions and recommendations of the study are not biased and are nonjudgemental, but they are supported by the data. The researcher ensured confirmability by playing back the recorded audio during interviews and the transcribed verbatim was retained for verification. Furthermore, the selected quotations from participants are included to allow the reader to judge the interpretations and credibility of the analysis.

Transferability was ensured by providing accurate and consistent information regarding the geographical background, context information of participants, sample, data collection, analysis and findings, as these allowed other researchers to assess how transferable the findings are.

## Results

The study revealed two main themes, danger alerts and emotional experiences of forewarning; communication with the baby, curbing their worries, protecting self and loved ones and the silence were subthemes that emerged from this paper.

## Presentation of findings

**FIGURE 1:**
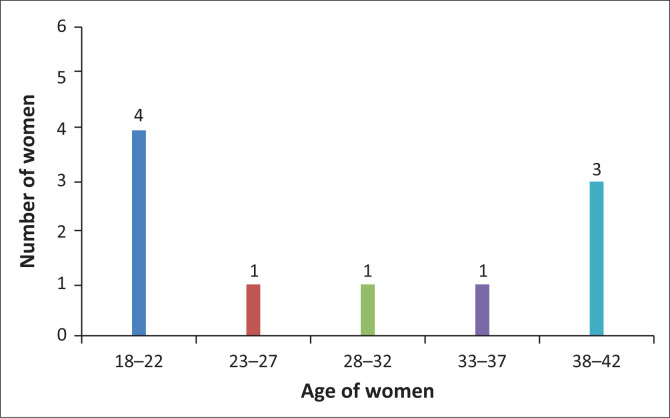
Participants’ age range.

**FIGURE 2:**
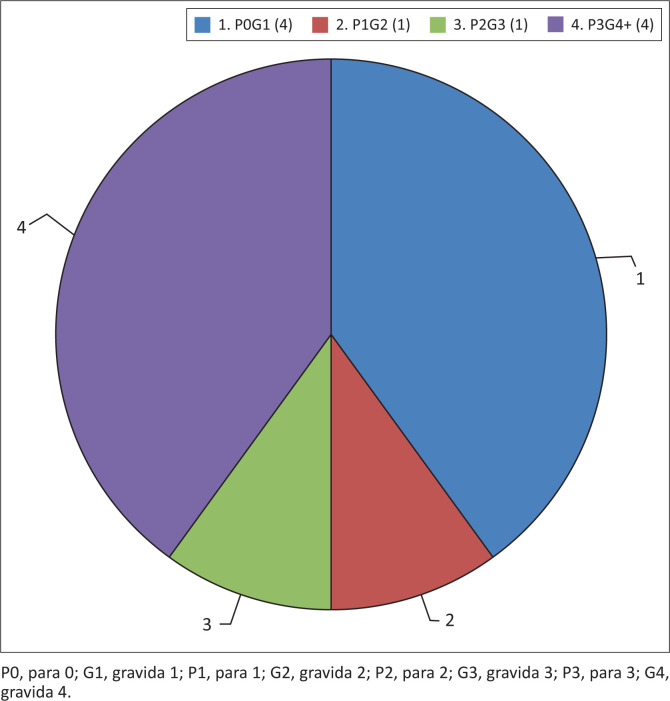
Participants’ parity.

The demographic information of the 10 participants was obtained from selected hospitals’ maternity registers and confirmed with the participants during the initial contact. Their ages ranged between 18 and 42 years, and the parity ranged from primigravida to para five at the time of study; this is indicated in Figure 1 and Figure 2.

From this study, two themes were found which presented various mind–body experiences of women who experienced IUFD before labour commences. These themes are discussed separately and interrelated with each other to reveal the experiences of women who experienced IUFD and have been identified based on the study objectives. Each theme consists of subthemes and is presented with the relevant quotes. Table 1 represents the themes, subthemes and extracts from the quotes.

**TABLE 1:** Themes, subthemes and extracts from the quotes.

Themes	Subthemes	Extract from quotes
1. Danger alerts	1.1. Forewarning	Stillness Painful umbilicus Reduced foetal movements Vomiting and headaches
	1.2. Communication with the baby	Using water to re-establish contact Changing position
2. Emotional responses	2.1. Resentment	Devastation Self-blame
	2.2. Curbing worries	Baby is just tired Baby is big
	2.3. Protecting self and loved ones	Requested more time Did not tell relatives
	2.4. The silence	Mumbled something Used silence Talked to each other

### Theme 1: Danger alerts

The danger alerts were the indications that participants experienced in the body while they were still at home, before contact with professional health care. According to participants, these danger alerts were feelings that informed them that something was not right in-utero before labour began. These signals informed them that the baby was not well in utero before labour began.

#### Subtheme 1.1: Forewarning

Throughout the pregnancy, a woman does feel some foetal movements and they are used to feeling those foetal movements. Getting used to the baby while still in utero helps women realise when there is a change in foetal movements. As per participants, these changes were referred to as a forewarning sign that something was not well in the womb and made them wonder if everything was still normal. Participants reported feeling painful umbilicus, vomiting, headaches and reduced foetal movements. Some reported that the baby did not kick as on other days; they thought that maybe if they could change their position, the baby might move. The findings reveal that some participants did not identify day-to-day changes, but the changes were only notifiable after some time. The following quotes attest thereto:

‘… I did not feel anything at the time; by month-end August, I started to feel that there were no foetal movements … my umbilicus was painful … my baby was still …’ (Participant 2, 21 years old, G1)

Similarly, some women narrated their ability to diagnose IUFD based on the absence of foetal movements. They said they could wake up during the night to listen to the baby’s movement, revealing that they could form strong bonds with the yet unborn baby, and were immediately aware if there were sudden foetal movement changes. The following quotes depict:

‘… I used to wake up at night because when the baby moved, but I slept all that night and woke early, and the first thing I felt was the stillness in my stomach …’ (Participant 3, 39 years old, G4)

‘… I spent the whole day alone trying to feel that there were movements, of which I felt nothing … but I knew that when I went to the clinic because I had a problem that I did not feel the baby kicks like the other days … and at the clinic, they did tell me that they could not hear anything …’ (Participant 1, 42 years old, G6)

#### Subtheme 1.2: Communication with the baby

Participants narrated the way they communicated with the baby as a warning sign that something was not right. The feelings of motionlessness in the stomach and lack of foetal movements when a woman is expecting movements were perceived as unusual behaviour. Therefore, when women actively sought contact with the baby by attempting different means of communication in the form of eating food, drinking, washing with water or changing position, no response came from the baby. After realising that there was no movement, one primiparous participant did not worry much and said:

‘… When I woke up in the morning I felt no movements … I just turned over to my other side of my stomach …. I thought that maybe the baby was just turning … I ended up just staying, but there were no foetal movements …’ (Participant 4, 18 years old, G1)

On the contrary, upon realising the lack of movements, some participants immediately sensed that something was wrong and tried to re-establish contact by eating, taking a bath and changing sleeping position. This is because of the bond that the women had already developed with their yet unborn child. They depicted the following:

‘… I woke up in the morning and I couldn’t feel the foetal movements … I went to the tshitanga [*outside hut used as a kitchen*] and stayed there until late … then I went to wash myself … still I could not feel any movements … I then ate food, thinking that my baby will move as usual … drank plenty of water … they [*midwives*] had said that I should drink cold water … still the baby never responded …’ (Participant 1, 42 years old, G6)

‘… There always used to be a movement in which I knew that everything is okay. One day I did not feel any movement; I then nudged and turned to another side … still there was no response; I then realised that my baby is in trouble. …’ (Participant 5, 35 years old, G3)

### Theme 2: Emotional responses

Upon realising that something was wrong, immediately a message was sent to participants’ minds which triggered their emotions as they curbed their worries, protected themselves and loved ones and the feeling of silence.

#### Subtheme 2.1: Resentment

Some participants revealed during the interviews that after hearing the news of the IUFD they felt devastated, like their hearts had been ripped apart, and said:

‘… When they showed me there was no heartbeat, I felt devastated … it was as if someone had ripped out my heart; my heart broke, and it’s never gone back right …’ (Participant 6, 19 years old, G1)

Study findings revealed that some participants were more inclined to blame themselves for the IUFD. The anger and the self-blame provide a powerful indicator of how women construed their emotional experiences. This has been substantiated by the following narrative:

‘… I couldn’t believe it, I felt angry, but the anger was directed at myself, thinking I have done something wrong, that maybe there must have been something that I did for this to happen …’ (Participant 3, 22 years old, G1)

#### Subtheme 2.2: Curbing the worries

Some participants narrated that they sometimes were in denial as they concealed their worries from others, not wanting to display their disappointment, but they consoled themselves by thinking that because they felt that they did not feel foetal movement, the baby might be tired. This was revealed by this participant:

‘… On arriving at the clinic, they checked me, and the nurse [*midwife*] told me she couldn’t feel anything [*no foetal heart rate*]; I told myself that the baby might just be tired …’ (Participant 7, 22 years old, G1)

Some participants thought that because the pregnancy was at its last trimester, the size of the baby had grown and the space inside the uterus had diminished, and the baby had settled in its position. The response was as follows:

‘… I thought because the baby was too big, there was not enough space to move around and it has settled down into a correct position; nothing is wrong …’ (Participant 1, 42 years old, G6)

Women normalised the lack of babies’ movement by curbing their worry for the baby and developing their interpretation, such as that the baby might be tired and is safe. Despite various evidence concerning normal foetal movements during the third trimester, there is a possibility that women were advised that the baby moves less towards the end of pregnancy. This made it difficult for women to distinguish decreased movements from lack of movement, because in the last trimester, these are considered normal.

#### Subtheme 2.3: Protecting self and loved ones

After hearing news of the IUFD, results revealed that some women kept it to themselves, unable to tell their relatives, some did not know how to tell their relatives, and needed to be given time. Furthermore, some participants wanted to tell their families about the loss of the baby but couldn’t because they were overwhelmed by emotions and not ready to talk. Therefore, nurses are the once who informed their relatives about IUFD:

‘overwhelmed by emotions, I was unable to phone my mother; I was unable to tell her telephonically; she was told by nurses [*midwives*] … I did not know how to tell her, and I was crying, unable to talk …’ (Participant 8, 18 years old, G1)

Other participants managed to hide their disappointment and refrained from disclosing the news to their relatives. One said:

‘… I didn’t tell my relatives anything; it was my secret … it was painful, but not yet time; I held on, just walking around without telling anyone … my relatives had to ask the nurses; that is when they were told.’ (Participant 5, 35 years old, G3)

The study revealed that participants took the news of IUFD as something which could be overturned, that maybe there could be a change from what the health professionals had said. They distanced themselves from the worst scenario – which was that the baby had died. Participants said:

‘… When they first told me that my baby is dead, I thought they were not serious …’ (Participant 4, 18 years old, G1)

‘… I told the doctor that before he could do anything, he must give me the whole day so that I could be sure that there is no longer any baby kicks … the doctor agreed, and I spent the day trying to feel that there were indeed no movements, of which I felt nothing …’ (Participant 5, 35 years old, G3)

#### Subtheme 2.4: The silence

The nonverbal response from the health professionals was one of the experiences that the women revealed. Participants revealed that healthcare professionals, after making a diagnosis of the IUFD, just mumbled something, used silence or only talked to each other, which made the women sense that there was something that was not right.

Some women were afraid to ask for clarity from the nurses upon sensing, through their maternal instincts, that something might be wrong; instead, they harboured the feelings within themselves and felt left out. Participants depicted these statements:

‘… I went for my 28 weeks’ scan, and the doctor mumbled something to the nurse and walked out of the room. … I didn’t know what he said, so thought I could see that something is not right …’ (Participant 6, 19 years old, G1)

‘… The silence worried me; it would have been better if the nurses at the clinic had talked during the examination there, explaining what they saw or what was puzzling them; I was supposed to have been told at the clinic that the baby is dead … at the examination room, the nurses there were communicating with each other without involving me in the discussion …’ (Participant 2, 21 years old, G1)

To the contrary, some participants narrated bravery and the need for clarity, standing up for what they believed about their maternal instincts; these are depicted in the following narrative:

‘… They didn’t say anything … everybody was silent, and I asked them “what’s going on, what’s happening?”… they didn’t say anything so I thought it could be serious, then I got out of bed and asked them again, looking at them, and they were all talking to each other but not to me … obviously if you are lying there and you’re expecting a baby and they don’t tell you anything, then you imagine all kinds of things …’ (Participant 3, 39 years old, G4)

## Discussion

The aim of the study was to explore the psychosomatic (mind–body) experiences of women who had an IUFD before labour commenced in rural areas of Limpopo province, South Africa. Therefore, the discussion of the findings will be based on the two identified themes, namely danger alerts and emotional responses.

### Theme 1: Danger alerts

The foetal movement is a psychosomatic (mind–body connection) subjective measure, mainly experienced and assessed by the mother. Thus, this qualitative study allowed all participating grieving mothers to narrate their lived psychosomatic experiences of the IUFD before labour commenced. The findings revealed that some women perceived the baby kicks and movements as the means of alert of baby wellness. Therefore, the moment they could not feel any movements, they became concerned, as it signalled that the baby was in danger before labour began. This is congruent with Lawn et al. (2015) and Trulsson and Radestad (2017), who state that a woman’s understanding of the pattern of movement is one method used to identify an unborn baby at risk. Similarly, Kleber Brom and Defare (2018) reported that 50% of women whose babies died in utero recognised a gradual decrease of foetal movements.

In agreement, Gerber-Epstein, Leichtentritt and Benyamini (2016) in a follow-up with 314 women three years after IUFD, stated that 23% said the lack of baby movements evoked their ‘very strong’ feeling that the baby was dead before a professional diagnosis was made. On the contrary, some women in this study did not perceive a decrease or cessation of baby movement as a sign that the baby was in danger. This is supported by Schott, Henley and Kohner (2016) when stating that the parity status of a woman can decrease her perception of foetal movement.

### Theme 2: Emotional responses

Lawn et al. (2015) indicated that IUFD encompasses several emotional dimensions of loss for women, and women suffer emotional pain. In agreement, this study’s participants narrated that upon hearing of the IUFD, they felt devastated and their hopes were shattered, as they were looking forward to having a baby. These findings correlate with Gerber-Epstein et al. (2016) when indicating that the greater the expectations of joy, the more painful it is when the hopes get shattered, because of grounded belief of fertility and the role of being a woman, because the loss undermines a woman’s worth and motherhood. Similarly, Lindgren, Malm and Rådestad (2016) indicate that the separation, going home empty-handed, letting go and leaving the baby at the hospital go against a mother’s biological instinct; this encounter is one for which they are unprepared, and it ruins the expectation of motherhood women felt during pregnancy.

Furthermore, this study reveals that women have feelings of self-blame for the death of a baby in utero, that maybe something that they ate or did led to the loss. These findings share the same sentiments with Adolfsson et al. (2010) when stating that women tend to blame themselves, thinking that maybe the loss has been through something that they did, ate or thought, and thus they grieve their profound loss. In agreement, Kilshaw et al. (2017) report that women who have lost their unborn babies were often sad, cried, yearned for their lost child and were inclined to blame themselves. Secondly, the feelings of self-blame, devastation, resentment and confusion were common to women who had IUFD.

All participating women in this study curbed their worries for the baby by normalising the absence of foetal movements, developing their interpretations and thinking that IUFD could be overturned. Schott, Henley and Kohner (2016) and the Saving Mothers Report (2017) concur with these findings, as they reported that approximately half the women who had IUFD had waited for up to 24 h or more without feeling baby movements and kicks before they contacted healthcare services.

These study findings revealed that women had difficulty with disclosing the loss to their family members. Similarly, Kilshaw et al. (2017) found that some women needed guidance on how to explain the death to the siblings and how to help them mourn.

This study revealed that the attending healthcare professionals did not communicate with or inform the women throughout the diagnosis of IUFD; instead, they were talking amongst themselves. Thus, women felt the silence from the attending healthcare professionals. In congruency, Gausia et al. (2017), Osman and Kersting (2017) and Kilshaw et al. (2017) found that some women reported blunt disclosures or silence from healthcare professionals during the ultrasound scans, as well as a lack of sympathy and compassion, but they insist that provision of good communication ensures that women receive high-quality, sensitive, consistent care and information from all staff during and after an IUFD, which is one of the most important elements of bereavement care for women during and following IUFD.

### Limitations

The study was limited to women above 18 years who had a NVD; the experiences of those who delivered through caesarean section were not captured.

Incorrect patient details at data capturing made it difficult to obtain patients’ files, as participants were followed up from the information provided.

## Conclusion

This qualitative study revealed that women are able to relate lack or decreased of foetal movement as the yardstick to diagnose IUFD or as a danger alert that the baby is not well before labour commences. This is depicted when women noticed the changes in the baby’s movement, because these movements are used as a means of daily communication with the baby, where lack of movements were a forewarning that the baby was in danger. Furthermore, the loss of the baby equally affected the emotional dimensions of women. Upon noticing that something was wrong, a message was sent to the women’s minds which activated their emotional responses. These included resentment, where they felt devastated; they curbed their worries by denying the loss; they protected themselves, and they felt silence from healthcare professionals, which pained them. This study’s findings revealed that all women who had IUFD went through the psychosomatic experiences before labour commenced. The study recommends further research regarding the kind of support needed for women after being informed of an IUFD.
